# In vitro dexamethasone suppression of cytokine production assay and plasma total and free cortisol responses to ACTH in healthy volunteers

**DOI:** 10.1038/s41598-025-26260-1

**Published:** 2025-12-03

**Authors:** Gladness Dakalo Nethathe, Jeremy Cohen, Jeffrey Lipman, Ronald Anderson, Karen Elizabeth Hay, Carel J. Pretorius, Charles Feldman

**Affiliations:** 1 School of Clinical Medicine, Faculty of Health Sciences, University of the Witwatersrand, Johannesburg, Johannesburg, South Africa; 2 Department of Anaesthesia and Perioperative Medicine, Royal Brisbane and Women’s Hospital, Level 4, Ned Hanlon Building, Butterfield Street, Herston, Brisbane, QLD 4029 Australia; 3 Faculty of Medicine, The University of Queensland, Brisbane, QLD Australia; 4 Intensive Care Services, Royal Brisbane and Women’s Hospital, Butterfield Street, Herston, , Brisbane, QLD 4029 Australia; 5 Jamieson Trauma Institute, Royal Brisbane and Women’s Hospital, Butterfield Street, Herston, Brisbane, QLD 4029 Australia; 6 University of Montpellier, Nimes University Hospital, Nimes, France; 7 The Clinical and Translational Research Unit of the Medical Oncology Centre of Rosebank Oncology Centre, Johannesburg, South Africa; 8 QIMR Berghofer Medical Research Institute, Brisbane, QLD Australia; 9 Department of Chemical Pathology, Pathology Queensland, Royal Brisbane and Women’s Hospital, Herston, Brisbane, QLD Australia

**Keywords:** Glucocorticoid sensitivity, Synthetic ACTH (1-24) stimulation, Total cortisol, Free cortisol, Adrenal insufficiency, Stress response, Endocrinology, Endocrine system and metabolic diseases, Adrenal gland diseases

## Abstract

Glucocorticoid sensitivity varies between individuals, influencing stress and inflammatory responses, however its relationship with the synthetic ACTH [1–24] stimulation test, is not well evaluated. To evaluate glucocorticoid sensitivity and plasma total cortisol (TC) and plasma free cortisol (PFC) responses to stimulation with 250 μg of synthetic ACTH [1–24]) in healthy adults. Prospective, single-centre observational study. Forty-eight healthy adults (24 females, 24 males) underwent basal and stimulated TC and PFC level measurements using liquid chromatography-tandem mass spectrometry. Peak cortisol values were defined as the highest TC or PFC at either 30- or 60-min. Glucocorticoid sensitivity was measured using the dexamethasone suppression of cytokine production (DSCP) assay, based on interleukin-6 (IL-6) and tumour necrosis factor-α (TNF-α) production from lipopolysaccharide-stimulated (LPS) monocytes. A whole blood sample was incubated with buffer, LPS, or LPS with dexamethasone (Dex). TNF-α and IL-6 concentrations were measured in the supernatants (ng/L). The ratio of cytokine levels between LPS + Dex and LPS indicated glucocorticoid sensitivity. Data from 45 participants were analysed. Peak TC levels ranged from 391 to 1570 nmol/L (median: 618 nmol/L, IQR: 555–729), while PFC levels ranged from 27 to 133 nmol/L (median: 62 nmol/L, IQR: 53–73). No significant associations were found between baseline or peak TC and PFC levels and glucocorticoid sensitivity ratios. Synthetic ACTH (1–24) test results did not predict glucocorticoid sensitivity. Males exhibited higher BMI (median: 26 vs. 22; *p* = 0.031), however no significant differences were observed in age, baseline PFC and TC levels between sexes. In this exploratory study, ACTH [1–24]—stimulated cortisol responses did not correlate with glucocorticoid sensitivity measured by the DSCP assay. Findings should be interpreted as preliminary and hypothesis-generating given current assay limitations.

**Supplementary Information:**

The online version contains supplementary material available at 10.1038/s41598-025-26260-1.

## Introduction

Glucocorticoid sensitivity (GS), which refers to the extent to which tissues or cells already capable of responding to glucocorticoids, exhibit changes in response to these hormones, plays a crucial role in regulating stress and inflammatory responses^1^. It is increasingly recognised that GS is a function of factors including circulating cortisol concentrations, tissue-specific factors such as glucocorticoid receptor (GR) expression, isoforms, polymorphisms, and local cortisol activation through 11β-HSD1 and 11β-HSD2^2,3^. These factors influence the genomic and non-genomic signalling pathways of GCs, contributing to variability in glucocorticoid effects across individuals and tissues, even when circulating levels are similar. Polymorphisms associated with increased or decreased GS have been linked to differences in metabolic risk, adiposity, and insulin resistance^2^.

Variability in GS has been observed among individuals, influencing clinical outcomes in conditions where the hypothalamic–pituitary–adrenal (HPA) axis is involved^1^. This variation appears to be at least partially ultradian in nature, aligning with the known pulsatile secretion of cortisol and the circadian acetylation of GRs, which modulate receptor activity throughout the day^2^.

Despite its clinical importance, the relationship between GS and glucocorticoid responsiveness, as measured by the standard short synacthen (synthetic ACTH (1–24), also known as tetracosactide) test, remains under-explored in healthy populations.

The synthetic ACTH (1–24) stimulation test is widely used to assess adrenal function by measuring cortisol production following an exogenous stimulus (synthetic ACTH (1–24)^4^. While traditionally focused on total cortisol (TC) levels, measurements of these levels obtained using immunoassays demonstrate significant variability^5^. Furthermore, TC responses to synthetic ACTH (1–24) are influenced by factors such as testing methodology and patient characteristics^6^. Immunoassays are more prone to cross-reactivity with other corticosteroids, which may lead to falsely elevated TC levels and liquid chromatography-tandem mass spectrometry (LC–MS/MS) is considered more accurate due to its high specificity and sensitivity^7^. With regards to patient factors, younger adults tend to have a more robust cortisol response to syntheticACTH (1–24) stimulation compared to older adults^8^. Total cortisol levels further depend on corticosteroid binding globulin (CBG), the primary carrier protein for cortisol in the blood. Thus factors that alter CBG levels, such as oestrogen therapy, pregnancy, or liver disease, can affect TC measurements without necessarily reflecting changes in plasma free cortisol levels (PFC)^9,10^. However, PFC is also influenced by several factors, including the binding affinity of corticosteroid binding globulin. Thus, interpreting PFC variability in healthy individuals provides an opportunity to examine its relationship to tissue-level glucocorticoid sensitivity, which may underlie inter-individual differences in HPA axis dynamics. Although our study does not directly assess these mechanisms, we acknowledge their potential to modulate PFC levels. Notably reference data for PFC in the healthy population are not well established and examining inter-individual variation in PFC responses may nonetheless provide insight into functional glucocorticoid exposure and sensitivity^11^.

Sex is increasingly recognised as a factor influencing TC and PFC^12,13^. Differences in steroid-binding proteins, the effect of interfering steroids on cortisol immunoassays and hormonal effects of oestrogen and progesterone, may dampen the cortisol response^13^. However, findings are conflicting, with some studies demonstrating higher stimulated cortisol responses in females, depending on the methodology^13–15^. Previous investigators have demonstrated higher peak TC and PFC levels in males following synthetic ACTH (1–24) stimulation, suggesting inherent sex-specific differences in adrenal function^12,16^. These findings are supported by Sofer and colleagues, who reported elevated PFC levels in men, and Roelfsema and colleagues, who observed higher mean cortisol levels in men under 50 years of age^12,16^. Emerging tissue-level biomarkers may offer improved resolution in assessing glucocorticoid status by better reflecting functional glucocorticoid activity at the cellular level. Clarke and colleagues advocate for the use of putative tissue-level biomarkers such as FKBP5 methylation, osteocalcin, and GDF-15 as proxies for glucocorticoid activity, though these remain investigational^2^.

Reduced sensitivity to glucocorticoids along with altered cortisol metabolism have been described as key pathophysiological factors in critical Illness-related corticosteroid insufficiency^17^. Although GS varies in chronic inflammatory states, its assessment and variability in critical illness using the in vitro monocyte dexamethasone suppression of cytokine production (DSCP) assay has only recently been reported with one study revealing a significant link between disease severity and GS in 21 patients with septic shock^18,19^. Although median tissue GS did not differ between patients with septic shock and control group participants in this study, patients with septic shock exhibited greater variability in GS with evidence of an association between illness severity and reduced GS^19^.

Furthermore, prior studies have demonstrated a positive correlation between PFC elevation following corticotrophin and illness severity^20–23^, and the PFC fraction is suggested by previous investigators to provide insights into individual variability in GS^19^. Given known variations in TC and PFC responses to corticotrophin, alongside differences in GS among healthy individuals, evaluating comparative normative data for the PFC response to corticotrophin and GS in healthy populations enhances the interpretation of adrenal function tests across various clinical contexts, as well as facilitating future comparative analyses with critically ill populations. We thus aimed to evaluate GS and the plasma total and free cortisol responses to the administration of synthetic ACTH (1–24) in healthy adults.

## Materials and methods

### Study design and participants

This prospective, single-centre observational study was conducted from the 2017 to 2020 and was approved by the Metro North Hospital Health Service Ethics Committee (HREC/15/QRBW/609). Written informed consent was obtained from all participants prior to inclusion in the study and the study was performed in accordance with the Declaration of Helsinki. Recruitment was conducted through the Royal Brisbane and Women’s Hospital (RBWH) and University of Queensland newsletters, as well as via social media posts. Exclusion criteria included age under 18 years, documented hypoadrenalism, or recent treatment with glucocorticoids (oral long-term use within 6 months or short-term use within the last four weeks). Although no participants were on corticosteroids, participants using inhaled or topical steroids, as well as those with well-controlled chronic conditions such as essential hypertension, were eligible for inclusion. In this study, one participant had essential hypertension, and another had asthma managed with salbutamol pro re nata. One participant declared oral contraceptive therapy.

### Study procedures

Participants underwent a medical assessment at study entry, including the collection of demographic data (age, sex, body mass index (BMI), medical history, and a physical examination. Once enrolment criteria were confirmed, a peripheral venous cannula was inserted for blood sampling. The standard short synacthen test (250 µg of synthetic ACTH (1–24), Novartis, NSW, Australia administered intravenously) was performed in an outpatient setting between 7:30 and 10:00 am. Blood samples were collected at baseline, and at 30- and 60- minutes post-synthetic ACTH (1–24) administration. Samples were processed for serum and ultra-filtrate, which were stored at –20°C until analysis. Plasma TC and PFC concentrations were measured using liquid chromatography-tandem mass spectrometry (LC–MS/MS)^24^. The highest cortisol value at either 30- or 60- minutes post-synthetic ACTH (1–24) was recorded as the peak cortisol concentration, with the difference between the peak and the baseline calculated as the cortisol increment.

### Glucocorticoid sensitivity assessment

Glucocorticoid sensitivity was assessed using the dexamethasone suppression of cytokine production (DSCP) test. This assay evaluates the ability of exogenous dexamethasone to suppress lipopolysaccharide (LPS)-stimulated monocyte production of IL-6 and TNF-α, by measuring the in vitro suppression of interleukin (IL)-6 and tumour necrosis factor (TNF-α) production from lipopolysaccharide (LPS)-stimulated leukocytes following dexamethasone administration. Lithium heparinised whole blood (1 mL) was separated into three portions and incubated in wells containing buffer (baseline), LPS with (LPS + Dex) or without dexamethasone (LPS) to a final concentration of 1000 nmol/L^18,19^. After a 3-h incubation, at 37°C the samples were centrifuged, to separate the plasma and the plasma was stored at –20°C until analysis on a Luminex analyser for IL-6 and TNF-α^18^. TNF-α and IL-6 production by monocytes was measured in the supernatants (ng/L). The buffer portion served as the baseline, LPS represented maximum cytokine stimulation, and LPS + Dex indicated glucocorticoid suppression. Results are expressed as a ratio. The ratio of change in cytokine levels from baseline between LPS + Dex and LPS indicated GS, with 95% reference intervals of 0.244–0.472 for IL-6 and 0.372–0.693 for TNF-α, with values outside these ranges indicating altered GS^18^. A lower ratio (values below these ranges) indicated increased GS (greater cytokine suppression), while a higher ratio (values above these ranges) suggested reduced sensitivity (less suppression)]^18,19^. The DSCP assay is a novel in vitro test, distinct from the standard dexamethasone suppression test used for Cushing’s syndrome^18^. Compared to previous methods like the thymidine assay, the DSCP assay offers superior sensitivity in detecting tissue glucocorticoid responsiveness^18^.

## Data analysis

### Statistical methods

Patient characteristics, cortisol and GS ratio measures of interest were summarised as median (IQR) and compared between sexes using Wilcoxon’s rank sum test. The distributions of variables of interest were assessed graphically using boxplots, histograms and scatterplots and outliers were identified and checked. Generalised linear mixed-effects models with a log-link and patient-level random intercepts were used to assess changes in cortisol levels over time. Overall associations between GS ratio measures, patient characteristics (age, sex BMI) and PFC and TC levels were tested in these models.

Peak TC and PFC values were derived as the maximum of the 30-min or 60-min values. Three missing values for peak PFC and one biologically implausible baseline value for PFC were replaced by the model-based predicted values. Correlations between IL-6 and TNF-α and cortisol measures were assessed using Pearson’s correlation coefficient and linear regression analyses. (

Statistical analyses were conducted using Stata software, version 18 (StataCorp. 2023. Stata Statistical Software: Release 18. College Station, TX: StataCorp LLC.)^25^.

## Results

### Safety and tolerability

Of the 48 healthy volunteers (24 males and 24 females) included in the study, eight (16.7%) reported minor self-limiting adverse effects following synthetic ACTH (1–24) administration. Four participants described experiencing a sensation of epigastric warmth immediately after injection and one reported a sensation of warmth spreading from head to toe. Two participants reported transient nausea lasting a few seconds and one developed a minor bruise at the cannula insertion site.

### Participant characteristics

Of 48 participants, 45 (aged 20–64 years) had sufficient data for inclusion in analyses. Summary statistics are shown in Table 1. The median BMI was significantly higher in males compared to females (26 vs. 22; *p* = 0.028). No significant differences in age were observed by sex. Baseline CBG levels, stratified by age, sex and BMI are provided in Supplementary Table 1.

**Table 1:** Summary statistics by group and sex

Variable	Total group (n = 45)	Female (n = 23)	Male (n = 22)	*p*-value
Age	33 (27–42)	32 (26–49)	33 (30–35)	0.94
Body mass index	24 (22–27)	22 (21–25)	26 (23–27)	0.028
Baseline plasma free cortisol (n = 44)	10 (6–17)	10 (8–17)	9 (5–17)	0.35
Plasma free cortisol at 30 min (n = 41)	46 (38–52)	41 (34–49)	49 (41–54)	0.074
Plasma free cortisol at 60 min (n = 42)	62 (53–71)	58 (52–70)	64 (54–71)	0.69
Baseline total cortisol	305 (225–391)	312 (269–458)	246 (206–391)	0.093
Total cortisol at 30 min (n = 44)	535 (493–574)	543 (493–820)	529 (493–553)	0.16
Total cortisol at 60 min	622 (555–729)	623 (568–874)	586 (546–690)	0.17
Ratio_Dex_IL6	0.31 (0.18–0.40)	0.30 (0.22–0.37)	0.35 (0.18–0.52)	0.31
Ratio_Dex_TNF-α	0.33 (0.26–0.43)	0.33 (0.25–0.39)	0.33 (0.27–0.48)	0.44

Variables are presented as median (interquartile range [IQR]). The number of participants for whom data was available is indicated where applicable (e.g., n = 41). TNF-α = Tumour Necrosis Factor-alpha, IL-6 = Interleukin-6, Dex = Dexamethasone, Ratio_Dex_IL-6 = Ratio of IL-6 after dexamethasone suppression, Ratio_Dex_TNF-α = Ratio of TNF-α after dexamethasone suppression.

### Cortisol response to synthetic ACTH (1–24)

**Fig. 1:**
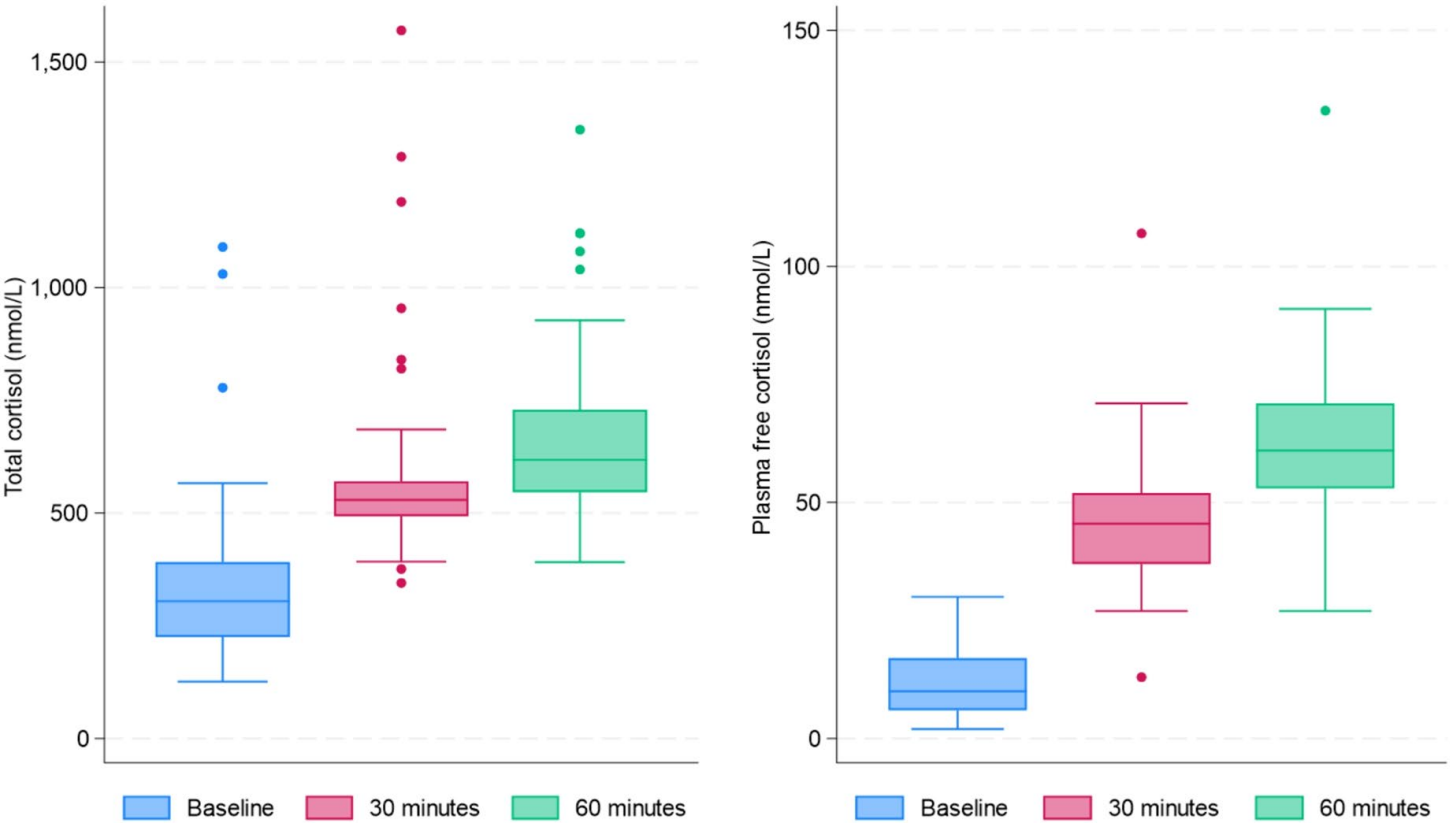
Boxplots showing the distribution of total cortisol and plasma free cortisol levels over time following administration of corticotrophin. Cortisol measurements are shown at baseline, 30 min, and 60 min post-baseline.

The distribution of TC and PFC levels over time following synthetic ACTH (1–24) stimulation is shown in Fig. 1. Peak total cortisol levels ranged from 391 to 1570 nmol/L (Median [IQR] 618 nmol/L, [555–729 nmol/L], while PFC ranged from 27 to 133 nmol/L, (Median [IQR] 62 nmol/L, [53–73 nmol/L]. No significant differences in baseline PFC, or TC levels were observed by sex. However, a trend was observed at 30 min post-synthetic ACTH (1–24) stimulation, where males had higher plasma free cortisol levels compared to females (Median [IQR]: 49 [41–54] vs. 41 [34–49] nmol/L; *p* = 0.074), although this difference did not reach statistical significance. In response to synthetic ACTH (1–24), participants exhibited an average fourfold increase in PFC levels from baseline to 30 min and an average 5.5 times increase from baseline to 60-min (Table 2). Corresponding increases for total cortisol were 1.7 and 1.9 times for total cortisol. There was no evidence for associations between cortisol measures and GS.

**Table 2:** Results of regression modelling of cortisol response to ACTH stimulation testing.

Variable	Category	RR^a^ (95% CI)	*p*-value	Marginal mean (95% CI)	Change from baseline mean (95% CI)
Plasma free cortisol					
Time	Baseline			11.7 (8.9–14.4)	
	30 min	4.0 (3.2–5.1)	< 0.001	46.8 (42.7–51)	35.1 (30.6–39.6)
	60 min	5.5 (4.4–6.9)	< 0.001	64.3 (59.3–69.3)	52.6 (47.4–57.8)
Ratio_Dex_IL-6		1.2 (0.8–1.8)	0.76		
Ratio_Dex_TNF-α		1.0 (0.6–1.6)	0.65		
Total cortisol					
Time	Baseline			356 (312–399)	
	30 min	1.7 (1.5–1.9)	< 0.001	600 (540–659)	244 (194–294)
	60 min	1.9 (1.7–2.1)	< 0.001	662 (598–725)	306 (254–358)
Ratio_Dex_IL-6		0.2 (− 0.4–0.7)	0.57		
Ratio_Dex_TNF-α		1.2 (0.7–2.1)	0.57		

^a^Estimates derived from generalised linear mixed effects models fitted with a log link and patient-level random intercept.

### Glucocorticoid sensitivity

A strong positive correlation was observed between the IL-6 and TNF-α ratio measures derived from the dexamethasone suppression tests (Pearson correlation coefficient = 0.82, *p* < 0.001).

There was considerable variation in ratio measures. Dex-IL-6 ratio values ranged from 0.02 to 0.78 with a mean (SD) and coefficient of variation (CoV) values of 0.32 (0.17) and 0.55 respectively. TNF-alpha ratio values ranged from 0.08 to 0.98 (mean: 0.34, SD:0.16; CoV: 0.47). Based on the cut-points for the IL-6 assay, 16/45 (36%) displayed increased GS, while for the TNF-α assay 30 (66%) were classified as having increased sensitivity. Decreased GS was observed for 10 (22%) for the IL-6 assay and one (2%) for the TNF-α assay. Only 6 (13%) participants had “normal” values on both assays.

#### Relationship between cortisol levels and glucocorticoid sensitivity

**Fig. 2:**
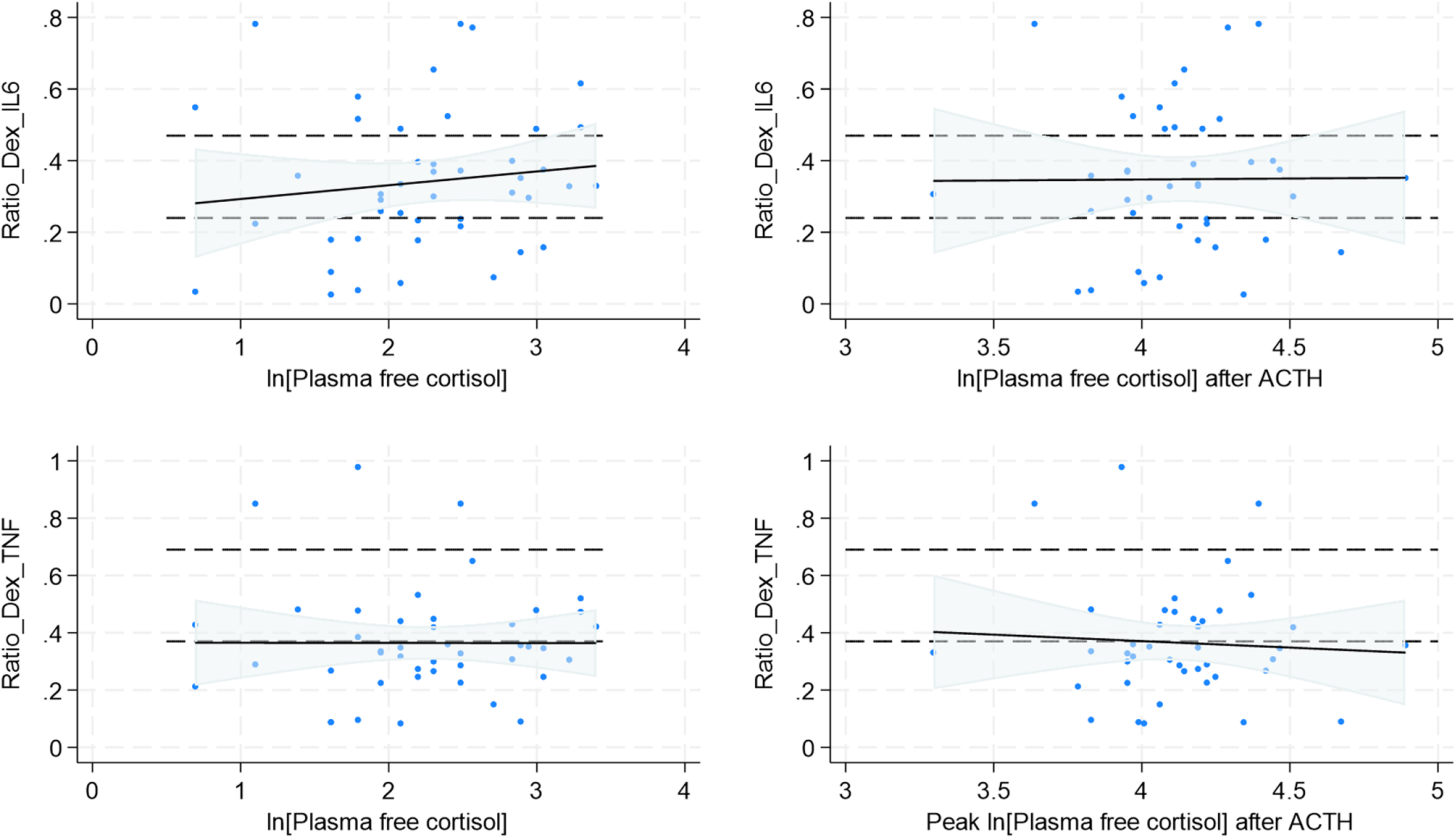
Relationship between Peak Plasma Free Cortisol (ln_peak_PFC) and Glucocorticoid Sensitivity (Ratios of TNF-α and IL-6). Scatter plots illustrate the relationships between the natural logarithm of peak plasma free cortisol (ln_peak_PFC) and glucocorticoid sensitivity, measured by the ratios of cytokine levels in dexamethasone-suppressed (LPS + Dex) versus LPS-stimulated conditions for TNF-α (Ratio_Dex_TNF-α) and IL-6 (Ratio_Dex_IL6). In both plots, each blue dot represents an individual data point. The red lines indicate the fitted linear regression lines, and the shaded grey areas show the 95% confidence intervals (CIs) for the fitted values. There is a wide variation in ratio values and no evidence of an association.

Scatterplots revealed wide dispersion of IL-6 and TNF-α ratio values around the best fitting linear regression lines (Fig. 2). On linear regression analyses, no significant associations were found between glucocorticoid ratio measures and cortisol measures (Table 3).

**Table 3:** Results of linear regression analyses assessing associations between glucocorticoid sensitivity ratio measures and plasma free cortisol and total cortisol following ACTH stimulation (n = 45).

Outcome	Variable	R^2^	Coefficient (95% CI)	*p*-value
IL-6 Ratio	Baseline PFC	0.059	0.006 (− 0.001–0.013)	0.11
	Peak PFC	0.003	0.001 (− 0.003–0.004)	0.73
	Proportion change PFC	0.024	0.00 (0.00–0.00)	0.31
	Baseline TC	0.005	0.00 (0.00–0.00)	0.63
	Peak TC	0.000	0.00 (0.00–0.00)	0.99
	Proportion change TC	0.016	− 0.02 (− 0.07–0.03)	0.41
TNF-α ratio	Baseline PFC	0.016	0.003 (− 0.004–0.01)	0.41
	Peak PFC	0.000	0.00 (− 0.003–0.003)	0.88
	Proportion change PFC	0.006	0.00 (− 0.01–0.01)	0.62
	Baseline TC	0.000	0.00 (0.00–0.00)	0.93
	Peak TC	0.015	0.00 (0.00–0.00))	0.43
	Proportion change TC	0.009	0.01 (− 0.03–0.06)	0.54

PFC, plasma free cortisol (per nmol/L); TC, total cortisol (per nmol/L).

Additional analyses examining associations between BMI and cytokine suppression ratios (IL-6 and TNF-α) showed no significant correlations (all *p* > 0.1),

## Discussion

The core finding of this study is the lack of a significant relationship between free and total cortisol responses to synthetic ACTH (1–24) and GS in healthy adults. Following synthetic ACTH (1–24) stimulation, there were no significant associations between peak TC or PFC levels and GS, as measured by IL-6 and TNF-α ratios.

The lack of a significant relationship between cortisol responses to synthetic ACTH (1–24) to GS observed in this study highlights findings by previous investigators that GS is associated with factors beyond circulating cortisol levels^26^. Although adrenal responsiveness to ACTH and peripheral GS are mechanistically distinct, tissue-level resistance may indirectly influence HPA axis dynamics. Reduced glucocorticoid signalling at tissue level may attenuate negative feedback, leading to increased ACTH drive and, over time, altered adrenal responsiveness^27,28^. Evidence for such tissue–central coupling has been reported primarily in obesity cohorts, where impaired glucocorticoid feedback correlates with elevated ACTH or enhanced adrenal output despite normal cortisol level^28,29^. This perspective aligns with the a hypothesised concept of an “extended hypothalamic–pituitary–adrenal-tissue axis,” whereby tissue-level glucocorticoid sensitivity can purportedly modulate central HPA axis regulation in specific populations. In this model, reduced peripheral glucocorticoid sensitivity results in impaired negative feedback and increased ACTH drive, potentially influencing adrenal responsiveness over time. This tissue-led feedback mechanism, demonstrating altered ACTH dynamics despite normal circulating cortisol has been demonstrated by various authors^28–30^. However, mechanistic data in healthy adults are limited, and our study was not designed to test central feedback. Thus, while speculative in healthy individuals, such mechanisms could contribute to inter-individual variation in ACTH-stimulated cortisol responses.

The considerable variability in GS indices observed among participants in this study likely reflects underlying biological heterogeneity in the general population. Glucocorticoid sensitivity is modulated by a variety of mechanisms, including the density and affinity of glucocorticoid receptors, co-chaperone proteins, and downstream signalling pathways that are not directly influenced by cortisol levels alone^26,31^.

Genetic polymorphisms in the glucocorticoid receptor gene are also known to significantly alter sensitivity to glucocorticoids, independent of cortisol concentrations^33^. Patients with glucocorticoid resistance or insensitivity can present with normal or elevated cortisol levels, suggesting that circulating cortisol is not always a reliable predictor (or cause) of glucocorticoid effects^34^. This is particularly evident in conditions such as glucocorticoid resistance syndrome, where therapeutic outcomes are not aligned with cortisol concentrations^35^. Furthermore, cortisol responses to stress, including ACTH stimulation, have been demonstrated to be highly variable across individuals, influenced by factors such as chronic stress, psychological conditions, and overall health status, without consistently correlating with GS^19^. Findings of the current study support the understanding that GS is a multifaceted phenomenon requiring a broader assessment framework that goes beyond simple cortisol measurements.

Male participants exhibited significantly higher body mass index (BMI) compared to females (26 vs. 22; *p* = 0.031), likely due to physiological differences in muscle mass, fat distribution, and hormonal factors such as higher testosterone levels^36–38^. No significant differences were observed in age between sexes. While baseline total cortisol levels tended to be higher in males, this difference did not reach statistical significance (*p* = 0.093).

### Independence of glucocorticoid sensitivity from cortisol measures and BMI

IL-6 and TNF-α ratio measures were independent of PFC and BMI in this population, suggesting that variations in these parameters do not significantly affect GS. Participants in this study had a normal BMI, indicating that baseline cortisol levels and BMI may not need to be heavily factored into GS assessments in adults within a normal BMI range, although this relationship might differ in overweight or obese populations^3^.

### Interrelationship between IL-6 and TNF-α suppression ratios

Another key finding of this study is the strong positive correlation between IL-6 and TNF-α suppression ratios (Pearson correlation coefficient = 0.82, *p* < 0.001), indicating that these ratio measures reflect a similar biological response in the DSCP assay. Both cytokines respond similarly when exposed to an inflammatory stimulus with their production being effectively suppressed by dexamethasone, highlighting a coordinated glucocorticoid effect on cytokine regulation. This high correlation reinforces the robustness of using these cytokine ratios in clinical and research settings to evaluate GS.

It is important to note the wide range of values for both ratios, with many falling outside the proposed reference range. This variability suggests that while the ratios capture a general suppression response, their ability to reliably differentiate between individuals with normal or impaired sensitivity may be limited. Despite this, the high correlation between IL-6 and TNF-α suppression suggests that these ratios still provide meaningful insights into GS, though further refinement of reference ranges may be necessary to enhance their discriminatory power in clinical and research settings.

### Study limitations and future directions

The relatively small sample size of the current study may have limited the power to detect more subtle associations between cortisol dynamics and GS. While we acknowledge the absence of a prospective power calculation, our sample size (n = 45) is comparable to previous similar exploratory studies^19^. Post-hoc power estimates indicated that with n = 45 (two-sided α = 0.05), the power to detect a correlation of r = 0.10 was 10% and r = 0.40 was 79%, indicating limited power for weak correlations but reasonable power for moderate correlations (NCSS 2020. PASS 2020: version 20.0.10. Kaysville, Utah: NCSS LLC.). The Pearson correlation coefficients between IL-6 and TNF-α ratios and log transformed peak PFC after ACTH stimulation in this study were 0.114 and 0.051 respectively, indicating a lack of correlation between GS and peak PFC. A larger sample size would be required in future studies to confirm these negligible or weak correlations. Future studies should explore mechanisms underlying observed sex differences and their potential clinical implications. explore these relationships in larger, more diverse populations and investigate additional factors such as genetic polymorphisms, receptor expression levels, and other markers of immune function. Additionally, while the DSCP assay has shown promise as a biologically grounded method for evaluating tissue-level glucocorticoid sensitivity, we acknowledge its limited standardisation in broader clinical settings. The reference ranges for GS (IL-6 and TNF-α suppression ratios) were derived from a cohort of 12 healthy volunteers, as described by Cardinal and colleagues^18^. While these ranges provide valuable insight, their derivation from a relatively small sample may impact the generalisability of these findings^18^.

Nonetheless, subsequent studies, such as Cohen and colleague’s investigation in septic shock, have replicated its use in larger and more diverse populations^19^. Our study applied the same methodology and laboratory procedures. However, we recognise that the DSCP assay remains incompletely validated, with normative data derived from small samples and limited reproducibility across laboratories. The absence of universally accepted thresholds or inter-laboratory standardisation constrains the strength of conclusions that can be drawn from our findings. We recognise that the lack of universally accepted thresholds for defining ‘normal’ versus ‘abnormal’ glucocorticoid sensitivity is a constraint. Standardisation across laboratories and larger reference datasets would strengthen the assay’s utility for comparative and clinical studies. For this reason, our results should be regarded as hypothesis-generating.

In addition, our study was limited by the absence of complementary biomarkers of glucocorticoid sensitivity such as glucocorticoid receptor expression, polymorphisms, or signalling intermediates^27^. As such, the DSCP assay should be viewed as reflecting one component of tissue-level glucocorticoid sensitivity, rather than providing a comprehensive assessment. Future studies combining functional assays with receptor-level and genetic analyses would provide a more integrated understanding^2^.

Despite these limitations, the DSCP assay currently represents one of the few available methods to assess functional tissue-level glucocorticoid effects in vitro, particularly in healthy populations where few alternatives exist.

Future studies with larger sample sizes are needed to further validate these reference ranges and confirm their applicability across broader populations and clinical settings.

## Conclusion

## Supplementary Information

Supplementary Information.

## Data Availability

Some or all datasets generated during and/or analysed during the current study are not publicly available but are available from the corresponding author on reasonable request.

## References

[1] Rohleder, N., Wolf, J. M. & Kirschbaum, C. Glucocorticoid sensitivity in humans-interindividual differences and acute stress effects. *Stress Amst Neth.* **6**(3), 207–222. 10.1080/1025389031000153658 (2003). 10.1080/1025389031000153658 13129814

[2] Wootton, E., Truong, Q., Pretorius, C. J., Balcerek, M. & Lazarus, S. A retrospective review of the short Synacthen test in Queensland hospitals. *Intern. Med. J.* **54**(9), 1515–1522. 10.1111/imj.16383 (2024). 38660891 10.1111/imj.16383

[3] Cohen, J., Ward, G., Prins, J., Jones, M. & Venkatesh, B. Variability of cortisol assays can confound the diagnosis of adrenal insufficiency in the critically ill population. *Intens. Care Med.* **32**(11), 1901–1905. 10.1007/s00134-006-0389-x (2006). 10.1007/s00134-006-0389-x 17019540

[4] Clark, A. J. L. & Weber, A. Adrenocorticotropin insensitivity syndromes. *Endocr. Rev.* **19**(6), 828–843. 10.1210/edrv.19.6.0351 (1998). 9861547 10.1210/edrv.19.6.0351

[5] Krasowski, M. D. et al. Cross-reactivity of steroid hormone immunoassays: Clinical significance and two-dimensional molecular similarity prediction. *BMC Clin. Pathol.* **14**(1), 33. 10.1186/1472-6890-14-33 (2014). 25071417 10.1186/1472-6890-14-33 PMC4112981

[6] Kushnir, M. M. et al. Liquid chromatography tandem mass spectrometry for analysis of steroids in clinical laboratories. *Clin. Biochem.* **44**(1), 77–88. 10.1016/j.clinbiochem.2010.07.008 (2011). 20627096 10.1016/j.clinbiochem.2010.07.008

[7] Verbeeten, K. C. & Ahmet, A. H. The role of corticosteroid-binding globulin in the evaluation of adrenal insufficiency. *J. Pediatr. Endocrinol. Metab.* **31**(2), 107–115. 10.1515/jpem-2017-0270 (2018). 29194043 10.1515/jpem-2017-0270

[8] Qureshi, A. C. et al. The influence of the route of oestrogen administration on serum levels of cortisol-binding globulin and total cortisol. *Clin Endocrinol.* **66**(5), 632–635. 10.1111/j.1365-2265.2007.02784.x (2007). 10.1111/j.1365-2265.2007.02784.x 17492949

[9] Dichtel, L. E. et al. Plasma Free cortisol in states of normal and altered binding globulins: Implications for adrenal insufficiency diagnosis. *J. Clin. Endocrinol. Metab.* **104**(10), 4827–4836. 10.1210/jc.2019-00022 (2019). 31009049 10.1210/jc.2019-00022 PMC6735741

[10] Sofer, Y. et al. Gender determines serum free cortisol: Higher levels in men. *Endocr. Pract.* **22**(12), 1415–1421. 10.4158/EP161370.OR (2016). 27540879 10.4158/EP161370.OR

[11] Dodd, A. et al. The effect of serum matrix and gender on cortisol measurement by commonly used immunoassays. *Ann. Clin. Biochem.* **51**(3), 379–385. 10.1177/0004563213514567 (2014). 24361991 10.1177/0004563213514567

[12] Clark, P. M., Neylon, I., Raggatt, P. R., Sheppard, M. C. & Stewart, P. M. Defining the normal cortisol response to the short Synacthen test: implications for the investigation of hypothalamic-pituitary disorders. *Clin. Endocrinol.* **49**(3), 287–292. 10.1046/j.1365-2265.1998.00555.x (1998). 10.1046/j.1365-2265.1998.00555.x 9861317

[13] Klose, M. et al. Factors influencing the adrenocorticotropin test: role of contemporary cortisol assays, body composition, and oral contraceptive agents. *J. Clin. Endocrinol. Metab.* **92**(4), 1326–1333. 10.1210/jc.2006-1791 (2007). 17244781 10.1210/jc.2006-1791

[14] Roelfsema, F. et al. Impact of age, sex and body mass index on cortisol secretion in 143 healthy adults. *Endocr. Connect.* **6**(7), 500–509. 10.1530/EC-17-0160 (2017). 28760748 10.1530/EC-17-0160 PMC5597974

[15] Annane, D. et al. Critical illness-related corticosteroid insufficiency (CIRCI): A narrative review from a Multispecialty Task Force of the Society of Critical Care Medicine (SCCM) and the European Society of Intensive Care Medicine (ESICM). *Intens. Care Med.* **43**(12), 1781–1792. 10.1007/s00134-017-4914-x (2017). 10.1007/s00134-017-4914-x 28940017

[16] Cardinal, J., Pretorius, C. J. & Ungerer, J. P. J. Biological and diurnal variation in glucocorticoid sensitivity detected with a sensitive *in vitro* dexamethasone suppression of cytokine production assay. *J. Clin. Endocrinol. Metab.* **95**(8), 3657–3663. 10.1210/jc.2009-2720 (2010). 20463096 10.1210/jc.2009-2720

[17] Cohen, J. et al. Glucocorticoid sensitivity is highly variable in critically Ill patients with septic shock and is associated with disease severity. *Crit Care Med.* **44**(6), 1034–1041. 10.1097/CCM.0000000000001633 (2016). 26963327 10.1097/CCM.0000000000001633

[18] Schein, R. M. H., Sprung, C. L., Marcial, E., Napolitano, L. & Chernow, B. Plasma cortisol levels in patients with septic shock. *Crit. Care Med.* **18**(3), 259–263. 10.1097/00003246-199003000-00002 (1990). 2302948 10.1097/00003246-199003000-00002

[19] Sam, S., Corbridge, T. C., Mokhlesi, B., Comellas, A. P. & Molitch, M. E. Cortisol levels and mortality in severe sepsis. *Clin Endocrinol.* **60**(1), 29–35. 10.1111/j.1365-2265.2004.01923.x (2004). 10.1111/j.1365-2265.2004.01923.x 14678284

[20] Annane, D. A 3-level prognostic classification in septic shock based on cortisol levels and cortisol response to corticotropin. *JAMA* **283**(8), 1038–1045. 10.1001/jama.283.8.1038 (2000). 10697064 10.1001/jama.283.8.1038

[21] Cohen, J. et al. Serial changes in plasma total cortisol, plasma free cortisol, and tissue cortisol activity in patients with septic shock: An observational study. *Shock* **37**(1), 28–33. 10.1097/SHK.0b013e318239b809 (2012). 21993448 10.1097/SHK.0b013e318239b809

[22] Quax, R. A. et al. Glucocorticoid sensitivity in health and disease. *Nat. Rev. Endocrinol.* **9**(11), 670–686 (2013). 24080732 10.1038/nrendo.2013.183

[23] Akalestou, E., Genser, L., Rutter, G. A. Glucocorticoid metabolism in obesity and following weight loss. *Front Endocrinol*. **11**. 10.3389/fendo.2020.00059. (2020) 10.3389/fendo.2020.00059 PMC7045057 32153504

[24] Grad, I. & Picard, D. The glucocorticoid responses are shaped by molecular chaperones. *Mol. Cell Endocrinol.* **275**(1), 2–12. 10.1016/j.mce.2007.05.018 (2007). 17628337 10.1016/j.mce.2007.05.018

[25] van Rossum, E. F. C. Polymorphisms in the glucocorticoid receptor gene and their associations with metabolic parameters and body composition. *Recent Prog. Horm. Res.* **59**(1), 333–357. 10.1210/rp.59.1.333 (2004). 14749509 10.1210/rp.59.1.333

[26] Raison, C. L. & Miller, A. H. When not enough is too much: the role of insufficient glucocorticoid signaling in the pathophysiology of stress-related disorders. *Am. J. Psychiatry.* **160**(9), 1554–1565. 10.1176/appi.ajp.160.9.1554 (2003). 12944327 10.1176/appi.ajp.160.9.1554

[27] Chrousos, G. P. Syndromes of glucocorticoid resistance. *Ann Intern Med.* **119**(11), 1113–1124. 10.7326/0003-4819-119-11-199312010-00009 (1993). 8239231 10.7326/0003-4819-119-11-199312010-00009

[28] Caglayan-Akay, E., Ertok-Onurlu, M. & Komuryakan, F. What factors drive gender differences in the body mass index? Evidence from Turkish adults. *J. Biosoc. Sci.* **55**(3), 538–563. 10.1017/S0021932022000190 (2023). 35509172 10.1017/S0021932022000190

[29] Lambert, E. et al. Gender differences in sympathetic nervous activity: Influence of body mass and blood pressure. *J Hypertens.* **25**(7), 1411–1419. 10.1097/HJH.0b013e3281053af4 (2007). 17563563 10.1097/HJH.0b013e3281053af4

[30] Schorr, M. et al. Sex differences in body composition and association with cardiometabolic risk. *Biol. Sex Differ.* **9**(1), 28. 10.1186/s13293-018-0189-3 (2018). 29950175 10.1186/s13293-018-0189-3 PMC6022328

[31] Lengton, R. et al. Variation in glucocorticoid sensitivity and the relation with obesity. *Obes. Rev.* **23**(3), e13401. 10.1111/obr.13401 (2022). 34837448 10.1111/obr.13401 PMC9285588

