# Supplementary material for: In vitro dexamethasone suppression of cytokine production assay and plasma total and free cortisol responses to ACTH in healthy volunteers
Source: Sci Rep. 2025 Dec 3;15:43127. doi: 10.1038/s41598-025-26260-1 (PMC12678605; doi:10.1038/s41598-025-26260-1)
Supplement: Supplementary file 1 — Supplementary Information. [file 41598_2025_26260_MOESM1_ESM.docx]

# Supplementary Table 1. Participant corticosteroid binding globulin (CBG) levels.

| **Participant Number** | **Age** | **Gender** | **Weight (kg)** | **Height (cm)** | **BMI (kg/m²)** | **CBG (µg/mL)** |
| --- | --- | --- | --- | --- | --- | --- |
| 1.0 | 35.1 | Male | 76.2 | 172.5 | 25.6 | 28.4 |
| 2.0 | 37.0 | Female | 58.4 | 158.0 | 23.4 | 36.9 |
| 3.0 | 26.0 | Female | 69.0 | 173.0 | 23.1 | 41.3 |
| 4.0 | 34.0 | Male | 63.9 | 178.0 | 20.2 | 27.4 |
| 5.0 | 26.0 | Female | 57.0 | 168.0 | 20.2 | 39.7 |
| 6.0 | 53.0 | Male | 82.0 | 172.0 | 27.7 | 21.5 |
| 7.0 | 32.0 | Female | 82.0 | 172.0 | 27.7 | 40.3 |
| 8.0 | 33.0 | Male | 63.0 | 175.0 | 20.6 | 24.6 |
| 9.0 | 31.0 | Female | 54.0 | 156.0 | 22.2 | 51.7 |
| 10.0 | 25.0 | Male | 90.0 | 188.0 | 25.5 | 28.2 |
| 11.0 | 25.0 | Male | 86.0 | 183.0 | 25.7 | 29.1 |
| 12.0 | 48.0 | Female | 67.6 | 163.0 | 25.4 | 30.8 |
| 13.0 | 20.0 | Female | 62.3 | 168.0 | 22.1 | 24.9 |
| 14.0 | 56.0 | Female | 86.0 | 160.0 | 33.6 | 27.7 |
| 15.0 | 32.0 | Male | 83.0 | 183.0 | 24.8 | 24.1 |
| 16.0 | 49.0 | Male | 85.0 | 178.0 | 26.8 | 26.4 |
| 17.0 | 25.0 | Female | 60.0 | 164.0 | 22.3 | 27.2 |
| 18.0 | 33.0 | Male | 94.0 | 185.0 | 27.5 | 28.7 |
| 19.0 | 33.0 | Male | 60.0 | 165.0 | 22.0 | 20.4 |
| 20.0 | 27.0 | Female | 48.0 | 151.0 | 21.1 | 23.7 |
| 21.0 | 24.0 | Female | 63.6 | 172.0 | 21.5 | 27.7 |
| 22.0 | 53.0 | Female | 60.0 | 165.0 | 22.0 | 20.4 |
| 23.0 | 32.0 | Male | 78.0 | 186.0 | 22.5 | 27.3 |
| 24.0 | 30.0 | Female | 48.0 | 150.0 | 21.3 | 20.4 |
| 25.0 | 33.0 | Female | 63.0 | 171.0 | 21.5 | 23.2 |
| 26.0 | 27.0 | Male | 77.3 | 172.0 | 26.1 | 32.1 |
| 27.0 | 60.0 | Male | 97.0 | 189.0 | 27.2 | 34.3 |
| 28.0 | 49.0 | Female | 68.0 | 168.0 | 24.1 | 32.5 |
| 29.0 | 26.0 | Female | 53.0 | 166.0 | 19.2 | 30.5 |
| 30.0 | 42.0 | Female | 113.0 | 165.0 | 41.5 | 27.0 |
| 31.0 | 30.0 | Male | 98.0 | 188.0 | 27.7 | 30.7 |
| 32.0 | 23.0 | Female | 72.0 | 175.0 | 23.5 | 24.5 |
| 33.0 | 64.0 | Female | 46.0 | 152.0 | 19.9 | 26.8 |
| 34.0 | 42.0 | Male | 92.0 | 186.0 | 26.6 | 27.3 |
| 35.0 | 30.0 | Male | 88.0 | 180.0 | 27.2 | 28.2 |
| 36.0 | 31.0 | Male | 82.0 | 188.0 | 23.2 | 30.0 |
| 37.0 | 27.0 | Male | 65.0 | 168.0 | 23.0 | 25.9 |
| 38.0 | 40.0 | Female | 65.0 | 162.0 | 24.8 | 27.1 |
| 39.0 | 26.0 | Female | 60.0 | 172.0 | 20.3 | 25.7 |
| 40.0 | 27.0 | Male | 93.0 | 189.0 | 26.0 | 26.8 |
| 41.0 | 37.0 | Male | 69.0 | 170.0 | 23.9 | 25.6 |
| 42.0 | 30.0 | Male | 85.0 | 175.0 | 27.8 | 24.6 |
| 43.0 | 51.0 | Female | 100.0 | 173.0 | 33.4 | 41.8 |
| 44.0 | 30.0 | Female | 57.0 | 164.0 | 21.2 | 30.4 |
| 45.0 | 35.0 | Male | 74.0 | 170.0 | 25.6 | 26.8 |
| 46.0 | 33.0 | Male | 69.0 | 172.0 | 23.3 | 31.4 |
| 47.0 | 59.0 | Female | 80.0 | 175.0 | 26.1 | 37.1 |
| 48.0 | 33.0 | Male | 80.0 | 175.0 | 26.1 | 26.5 |
